# Improving Psychological Well-Being and Quality of Life in Rheumatoid Arthritis-Associated Interstitial Lung Disease Through Exercise and Pulmonary Rehabilitation: A Narrative Review

**DOI:** 10.3390/healthcare14050657

**Published:** 2026-03-05

**Authors:** Adithan Ganesh, Shivani Mishra, Grace W. Hwang, Shenar Dinkha, Emilie Chan

**Affiliations:** 1 Department of Medicine, Icahn School of Medicine at Mount Sinai, NYC Health + Hospitals/Elmhurst, Queens, NY 11373, USA; 2 Institute for Excellence in Health Equity, NYU Grossman School of Medicine, New York, NY 10016, USA; 3 Department of Medicine, Penn State College of Medicine, Pennsylvania State University, Hershey, PA 17033, USA; 4 Department of Medicine, University of Arizona College of Medicine–Tucson, Tucson, AZ 85721, USA

**Keywords:** rheumatoid arthritis, interstitial lung disease, pulmonary rehabilitation, quality of life, psychological health

## Abstract

**Background**: Rheumatoid arthritis-associated interstitial lung disease (RA-ILD) is a significant extra-articular manifestation of rheumatoid arthritis that contributes to morbidity, functional limitation, exertional dyspnea, fatigue and reduced quality of life. Current pharmacologic therapies address inflammatory and fibrotic pathways but do not target deconditioning, ventilatory inefficiency, skeletal muscle dysfunction or the high burden of anxiety, depression, impaired emotional well-being and reduced daily functioning. **Objective**: To synthesize the evidence supporting the use of structured exercise and pulmonary rehabilitation (PR) on functional, psychological and quality of life outcomes in RA-ILD patients by integrating findings from RA-ILD cohorts, interstitial lung disease (ILD) PR trials, and rheumatoid arthritis (RA) exercise interventions. **Methods**: A structured narrative review was conducted using PubMed, Scopus, and Web of Science (1990–2025). Of the 1240 identified records, 32 studies met inclusion criteria, comprising 4 RA-ILD observational cohorts, 17 ILD PR trials and 11 RA exercise trials. No randomized controlled trials specifically evaluating PR in RA-ILD were identified. Available evidence was extrapolated from studies in general ILD and RA populations. Mechanistic and physiological literature was included to contextualize findings. **Results**: RA and ILD cohorts demonstrated markedly reduced six-minute walk distance, impaired diffusing capacity, exertional desaturation, fatigue, high anxiety and depression, and diminished daily function. Across seventeen PR trials, patients with idiopathic, autoimmune-associated, and fibrotic ILD showed improvements in exercise capacity, ventilatory efficiency, dyspnea, fatigue, psychological distress, emotional well-being, and health-related quality of life. Eleven RA exercise studies demonstrated improved aerobic capacity, strength, lean mass, fatigue, psychological outcomes (including anxiety), and function, with no increase in disease activity. **Conclusions**: Evidence from ILD PR and RA exercise literature suggest that structured rehabilitation has the potential, alongside pharmacological therapy, to address functional limitation, dyspnea, fatigue and psychological distress and overall quality of life in RA-ILD, though disease-specific trials are needed.

## 1. Introduction

Rheumatoid arthritis (RA) is a systemic autoimmune disease affecting approximately 1% of adults worldwide and characterized by chronic synovitis, progressive joint destruction, and multi-organ involvement [1,2]. Interstitial lung disease (ILD) represents one of the most serious extra-articular manifestations of RA, accounting for substantial morbidity and mortality. Clinically apparent RA-ILD occurs in 7–10% of patients, while high-resolution computed tomography (HRCT) studies indicate that 20–30% may have subclinical ILD depending on RA duration, smoking status, and genetic predisposition [3,4].

The major HRCT patterns—usual interstitial pneumonia (UIP) and nonspecific interstitial pneumonia (NSIP), carry different prognostic implications. UIP confers more rapid physiologic decline, increased fibrosis, worse survival, and greater exertional desaturation than NSIP [3,4], suggesting that exercise interventions in this subgroup may require closer monitoring of oxygen saturation, although current evidence does not indicate a differential safety profile. Physiologically, RA-ILD produces restrictive lung defects, impaired diffusing capacity (DLCO), ventilatory inefficiency, and exertional hypoxemia. Beyond pulmonary limitation, RA contributes systemic inflammation, muscle wasting, joint deformities, chronic pain, fatigue, and reduced mobility, all of which interact to impair exercise capacity [5,6].

RA-ILD observational studies consistently demonstrate markedly lower 6-min walk distance (6MWD), impaired gas exchange, and reduced daily physical activity compared with RA patients without ILD [5,6,7,8]. Dyspnea is often severe and may exceed what is predicted by spirometry alone, reflecting contributions from neuromechanical dissociation, impaired ventilatory efficiency, and altered central perception of breathlessness. Fatigue-a pervasive symptom reported by more than 70% of RA patients-is amplified by systemic inflammation, hypoxemia, sleep disturbance, and physical deconditioning [9,10].

Psychological comorbidities are highly prevalent. Anxiety and depression are closely correlated with dyspnea severity, fatigue, and functional impairment in ILD and RA-ILD cohorts. A maladaptive dyspnea-anxiety-deconditioning cycle often emerges, wherein fear of breathlessness leads to activity avoidance, accelerating deconditioning and worsening symptoms [11].

Pharmacologic therapy-including glucocorticoids, immunosuppressants, and antifibrotic agents-may slow RA-ILD progression but does not target deconditioning, impaired ventilatory efficiency, skeletal muscle dysfunction, or psychological distress [12,13,14,15,16]. Pulmonary rehabilitation (PR), recommended by ATS/ERS guidelines for ILD management, improves exercise tolerance, dyspnea, fatigue, mood, and health-related quality of life across fibrotic and non-fibrotic ILD [17,18,19,20,21,22,23,24]. Similarly, structured exercise interventions for RA-including aerobic, resistance, and mind-body programs-consistently improve physical function, fatigue, strength, and emotional well-being without increasing disease activity [25,26,27,28,29,30,31].

Because RA-ILD exists at the intersection of restrictive lung disease, chronic inflammation, sarcopenia, joint pathology, dyspnea, and psychological distress, a tailored exercise-based intervention may address multiple domains simultaneously. Yet no randomized interventional trials have evaluated rehabilitation specifically in RA-ILD. This review integrates RA-ILD observational evidence, ILD PR trial outcomes, and RA exercise literature to provide a mechanistically grounded and clinically relevant rationale for implementing structured rehabilitation in RA-ILD.

## 2. Materials and Methods

A structured narrative review methodology was used to synthesize evidence related to functional impairment, psychological burden, and rehabilitative interventions in rheumatoid arthritis-associated interstitial lung disease (RA-ILD). Where available, systematic reviews and randomized controlled trials were prioritized; smaller observational and mechanistic studies were included to contextualize physiologic and clinical findings. This approach allowed integration of heterogeneous evidence sources and mechanistic insights.

### 2.1. Search Strategy

Comprehensive searches were conducted using PubMed, Scopus, and Web of Science from January 1990 to January 2025. Search terms included combinations of: “rheumatoid arthritis”, “interstitial lung disease”, “RA-ILD”, “pulmonary rehabilitation”, “exercise therapy”, “aerobic training”, “resistance training”, “dyspnea”, “fatigue”, “psychological distress”, “ventilatory efficiency”, “skeletal muscle” and “inflammation”.

Boolean operators and MeSH terms were incorporated. Broad terms such as ‘inflammation’ were used only in combination with rheumatoid arthritis, interstitial lung disease, and exercise-related terms using Boolean AND operators, primarily to capture mechanistic literature; these terms were not used as standalone searches and did not independently contribute to the overall search yield. Reference lists of included articles and major guideline statements were manually screened.

### 2.2. Study Selection

Inclusion criteria:

1. Adult participants (≥18 years).
2. Diagnosed RA-ILD, ILD of any subtype, or RA.
3. Evaluation of exercise capacity, dyspnea, fatigue, psychological outcomes, strength, or structured rehabilitation/exercise.
4. Study design: randomized controlled trials, prospective or retrospective cohorts, or cross-sectional analyses.
5. Sample size ≥ 5
6. English language.

Exclusion criteria included studies focusing solely on pharmacologic interventions without functional or symptomatic outcomes. A minimum samples size of ≥5 was selected to exclude isolated case reports while allowing inclusion of early physiologic and observational studies in this relatively uncommon disease population.

### 2.3. Screening Process

Searches identified 1240 records. After deduplication, 1033 titles and abstracts were screened, yielding 940 potentially relevant abstracts. Sixty full texts were reviewed; 32 studies met criteria:

- 4 RA-ILD observational studies
- 17 ILD pulmonary rehabilitation trials
- 11 RA exercise interventions

Mechanistic, physiologic, and guideline literature was incorporated to contextualize findings.

### 2.4. Data Extraction and Synthesis

Data elements extracted included study design, sample characteristics, intervention protocols, duration, and key functional, physiologic, psychological, and quality-of-life outcomes. Narrative synthesis was used to integrate findings across domains, with emphasis on mechanistic plausibility and cross-population applicability.

All included studies were reviewed in full by the authors. Artificial intelligence tools (ChatGPT versions 4.0, 5.0, and 5.1; OpenAI) were used solely to assist with language editing and textual conciseness. All data extraction, interpretation, and synthesis were performed by the authors and manually verified against the original source manuscripts. The authors take full responsibility for the accuracy and integrity of the content.

## 3. Results

### 3.1. Clinical and Physiologic Findings from RA-ILD Observational Cohorts

Table 1 summarizes key observational studies characterizing the clinical trajectory, physiologic decline, and functional burden of rheumatoid arthritis-associated interstitial lung disease (RA-ILD). Across the included studies (sample sizes 38–679), RA-ILD consistently emerged as a progressive, high-risk phenotype. Longitudinal cohort data demonstrated measurable annual declines in FVC and DLCO, with the UIP radiologic pattern associated with the steepest deterioration and highest mortality. Population-based studies further highlighted the substantial comorbidity burden in RA-ILD, particularly COPD and cardiovascular disease-along with approximately twofold higher mortality compared with RA without ILD. Additional work examining dyspnea, functional status, and psychological health showed strong correlations between dyspnea severity, reduced 6-min walk performance, and depressive symptoms. Finally, improvements in real-world physical activity following RA disease-modifying therapy reinforce the link between systemic inflammation and functional behavior. Together, these data define the natural history of RA-ILD and provide the physiologic rationale for exploring structured exercise and pulmonary rehabilitation in this population.

### 3.2. Effects of Pulmonary Rehabilitation in ILD 

Table 2 synthesizes evidence from studies evaluating pulmonary rehabilitation (PR) and exercise training across a spectrum of ILD phenotypes, including IPF, CTD-ILD, mixed ILD, and advanced ILD. Where subgroup analyses were included, results were included in the table. Sample sizes varied from small mechanistic cohorts (<30 participants) to large inpatient rehabilitation datasets (>400 participants), in addition to meta-analytic evidence. Despite heterogeneity in ILD subtype and program duration, nearly all studies demonstrated clinically meaningful improvements in exercise tolerance, dyspnea, mood disorders and health-related quality of life following PR. Psychological outcomes were commonly assessed using validated instruments such as the Hospital Anxiety and Depression Scale (HADS), Beck Depression Inventory (BDI) and mental health domains of the SF-36. Randomized trials in IPF showed short-term gains in functional capacity, while observational cohorts demonstrated that PR completers experienced sustained improvements and, in some cases, better long-term outcomes. Predictors of PR benefit included lower baseline functional impairment and fewer comorbidities, while mechanistic studies identified substantial exertional oxygen requirements that can be safely titrated during training. Importantly, CTD-ILD cohorts-including diseases closely related to RA-ILD-also showed significant improvements in 6MWD, dyspnea, and fatigue, supporting the applicability of PR to autoimmune-mediated ILD. Collectively, these findings establish PR as a safe, feasible, and efficacious intervention across ILD phenotypes relevant to RA-ILD.

### 3.3. Effects of Exercise Interventions in Rheumatoid Arthritis

Table 3 summarizes randomized controlled trials, mechanistic studies, and meta-analyses evaluating structured exercise training in rheumatoid arthritis (RA). On detailed review of the RA exercise trials, most studies did not explicitly report exclusion or inclusion criteria related to ILD or other pulmonary pathology, and systematic pulmonary phenotyping was generally not performed. Across 20-week to 2-year interventions, resistance training, high-intensity training, aerobic exercise, yoga, and Tai Chi were shown to be safe, well tolerated, and beneficial. High-intensity resistance training improved muscle strength, lean mass, and functional capacity without exacerbating disease activity. Aerobic exercise and combined training programs consistently reduced fatigue, improved cardiorespiratory fitness, and enhanced physical function. Mind-body interventions such as yoga and Tai Chi improved pain, stiffness, and quality of life. Meta-analytic evidence further demonstrated that neither aerobic nor resistance exercise increases RA disease activity. A mechanistic trial additionally showed that moderate-to-high intensity exercise reduced peripheral regulatory T-cell populations in older adults with RA, suggesting immunologic modulation beyond functional benefits. The evidence strongly supports exercise as a safe and effective adjunct to medical therapy in RA, with potential translational relevance for RA-ILD patients who suffer from systemic inflammation, reduced muscle mass, and diminished exercise tolerance.

## 4. Discussion

Rheumatoid arthritis-associated interstitial lung disease (RA-ILD) represents one of the most disabling and life-limiting extra-articular manifestations of rheumatoid arthritis. Despite the high morbidity associated with RA-ILD, evidence-based non-pharmacologic management strategies remain underutilized, and no formal guidelines currently address the role of exercise training or pulmonary rehabilitation (PR) in this population. The present review integrates three distinct but highly complementary bodies of evidence—studies describing the natural history of RA-ILD, clinical trials of PR in broader ILD cohorts, and randomized trials of structured exercise training in RA, to establish a mechanistic and empirical foundation supporting the use of PR for individuals with RA-ILD.

### 4.1. Integrating Evidence Across RA-ILD, ILD Rehabilitation, and RA Exercise Studies

Studies of RA-ILD consistently demonstrate a pattern of progressive pulmonary impairment, particularly among patients with a UIP pattern on imaging. Longitudinal cohort data show continuous declines in forced vital capacity (FVC) and diffusing capacity for carbon monoxide (DLCO), with annual declines comparable to those observed in idiopathic pulmonary fibrosis (IPF) [32]. Population-level analyses confirm that RA-ILD confers a markedly increased mortality risk, often exceeding that seen in seropositive RA without lung involvement [33]. Beyond physiologic decline, RA-ILD is associated with substantial symptomatic burden reflected in exertional dyspnea, reduced six-minute walk distance (6MWD), impaired daily function, and high rates of depression and fatigue [34,35]. These findings collectively characterize RA-ILD as a multisystem disease in which pulmonary restriction, muscular dysfunction, physical inactivity, and distressing respiratory symptoms are deeply intertwined.

In contrast to the limited RA-ILD-specific literature, the evidence base for PR in other forms of ILD is extensive. Across a wide range of ILD subtypes, including IPF, fibrotic nonspecific interstitial pneumonia (NSIP), mixed ILD cohorts, and connective tissue disease-associated ILD (CTD-ILD), PR consistently yields clinically meaningful improvements in exercise capacity, dyspnea, and health-related quality of life. Early work by Holland et al. [36] demonstrated short-term gains in 6MWD, symptom burden, and health status following structured exercise training in ILD patients, while subsequent studies identified predictors of PR responsiveness and confirmed that individuals with ILD experience benefits comparable to or exceeding those seen in COPD [37]. Prospective cohort studies confirm that PR is associated not only with functional improvements but also with durable clinical benefits, including better long-term symptom control and potentially reduced mortality [39]. More recent randomized controlled trials in IPF and CTD-ILD support the reproducibility of these improvements across diverse ILD phenotype [40,43]. The 2021 Cochrane review synthesizing ILD rehabilitation research further reinforces that PR is effective, safe, and clinically meaningful for patients with fibrotic lung disease [38].

Complementing the ILD rehabilitation literature, RA exercise trials provide strong evidence that systemic exercise interventions-including resistance training, aerobic conditioning, yoga, Tai Chi, and high-intensity training, are safe and confer broad physiologic and psychological benefits to RA patients. High-intensity resistance training improves muscle strength, lean body mass, and functional performance without exacerbating joint disease activity [53,58]. Aerobic exercise reduces fatigue, enhances aerobic capacity, and improves quality of life, while meta-analyses confirm that exercise does not precipitate RA flares or worsen inflammatory markers [24,56]. Importantly, exercise-induced immunologic effects have also been demonstrated, such as reductions in peripheral regulatory T-cell populations and altered inflammatory profiles following combined aerobic and resistance interventions [57].

These domains of research—RA-ILD natural history, ILD pulmonary rehabilitation, and RA exercise trials, form a strong, convergent evidence base indicating that patients with RA-ILD possess both the physiologic need for and the biological capacity to benefit from structured exercise interventions.

### 4.2. Pathophysiologic Rationale

RA-ILD reflects the convergence of pulmonary restriction, systemic inflammation, musculoskeletal dysfunction, and psychological distress. Fibrotic remodeling reduces lung compliance and increases the elastic load on the respiratory system, leading to heightened neural respiratory drive and neuromechanical dissociation, both well-established contributors to exertional dyspnea in ILD [23,36,59]. These abnormalities cause dyspnea at disproportionately low workloads, mirroring physiologic patterns described in IPF and other fibrotic lung diseases [12,22].

Exercise training targets these physiologic derangements. Aerobic and resistance exercise improve ventilatory efficiency by enhancing mitochondrial density, oxidative metabolism, and peripheral muscle oxygen extraction-mechanisms repeatedly demonstrated in RA cohorts and ILD rehabilitation programs [5,27,28,36,37,60]. Resistance training reverses RA-associated muscle catabolism mitigates reductions in muscle cross-sectional area, and improves functional capacity [6,7,53,58]. Aerobic conditioning improves endothelial function and capillary density, further reducing lactate accumulation and ventilatory demand during exertion.

Systemic inflammation in RA accelerates muscle wasting and impairs bioenergetic efficiency, partly mediated by elevated TNF-α and IL-6 signaling [1,2,61]. Exercise counteracts these effects by activating anabolic pathways and reducing inflammatory cytokine activity, including demonstrated reductions in regulatory T-cell populations following combined aerobic and resistance interventions [57]. These physiologic improvements translate into enhanced work capacity, reduced perceived effort, and better tolerance for physical exertion.

**Figure 1:**
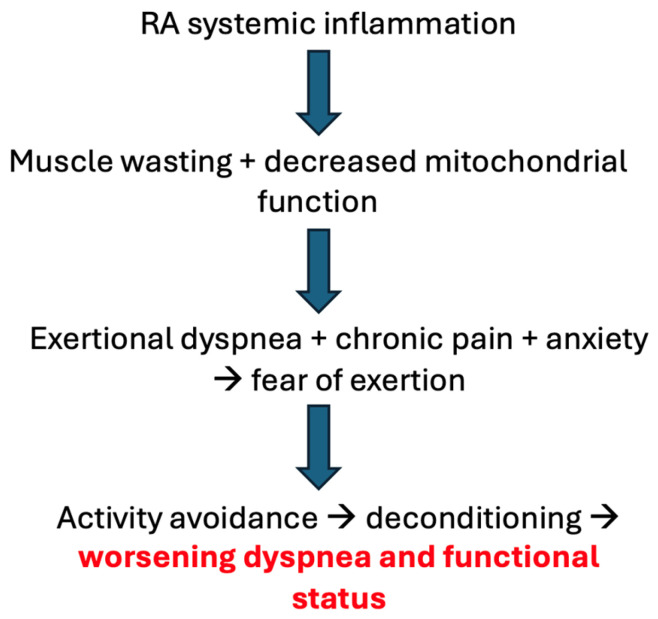
Interaction of inflammatory, muscular, pulmonary, and physiological mechanisms producing functional impairment in RA-ILD.

Psychological factors shape the perception of breathlessness through cortical and limbic integration of sensory input, threat appraisal, and affective response [9,10,11,62]. Both PR and structured exercise reduce anxiety and catastrophizing, altering central processing of dyspnea and improving symptom appraisal. Improvements in depression and fatigue-highly prevalent in RA and ILD-are well documented across multiple exercise trials [8,19,31]. Collectively, these mechanisms (Figure 1) support a strong biological rationale for PR in RA-ILD.

### 4.3. The Dyspnea-Anxiety-Deconditioning Cycle

The cyclical interaction of dyspnea, anxiety, and deconditioning plays a central role in functional decline in RA-ILD. Exertional dyspnea prompts anticipatory fear and avoidance of activity, leading to skeletal muscle atrophy, reduced oxidative capacity, and impaired ventilatory efficiency-patterns described in ILD and RA cohorts alike [20,27,35,36]. Reduced muscle mass and strength further heighten ventilatory demand during exercise, perpetuating a feedback loop in which dyspnea becomes progressively more limiting.

RA-specific factors-including fatigue, pain, morning stiffness, and poor sleep-exacerbate this cycle and reduce baseline activity levels [8,27]. In ILD, PR consistently demonstrates improvements in anxiety, depressive symptoms, self-efficacy, and coping, breaking this cycle through graded exposure and supervised conditioning [36,37,38,39,40,45,50]. RA exercise trials similarly demonstrate reductions in fatigue and improvements in mood, functional capacity, and self-efficacy [24,27,28,29].

This shared pattern between RA and ILD literature underscores the relevance of PR in RA-ILD, where dyspnea, fear-avoidance, and deconditioning are tightly interwoven physiological and behavioral processes.

### 4.4. Practical Implementation

Optimal implementation of PR in RA-ILD requires coordination between pulmonology, rheumatology, and rehabilitation specialists. Baseline assessment should include pulmonary function testing, exertional oximetry, RA disease activity evaluation, fatigue scales, and screening for anxiety and depression-domains emphasized in ILD PR guidelines and RA exercise trials [16,17,23,25].

Exercise prescriptions should be individualized and initiated at low to moderate intensity with gradual progression. Interval training can be particularly useful for patients with severe dyspnea or marked desaturation, consistent with approaches used in advanced ILD cohorts [42]. Resistance training programs should incorporate joint-protection strategies and allow for RA disease fluctuation, drawing from successful RA strength-training trials demonstrating safety and high efficacy [52,53,58]. Standard pulmonary rehabilitation equipment may require modification for patients with RA-related joint deformities. Recumbent bicycles can reduce axial loading and hip or spinal stress, while arm ergometers or semi-recumbent devices may be preferable for individuals with lower-extremity limitations. For patients with hand or wrist deformities who cannot grip standard handles, modified grips, strap-based interfaces, or forearm-supported ergometers may facilitate safe participation while minimizing joint strain.

**Figure 2:**
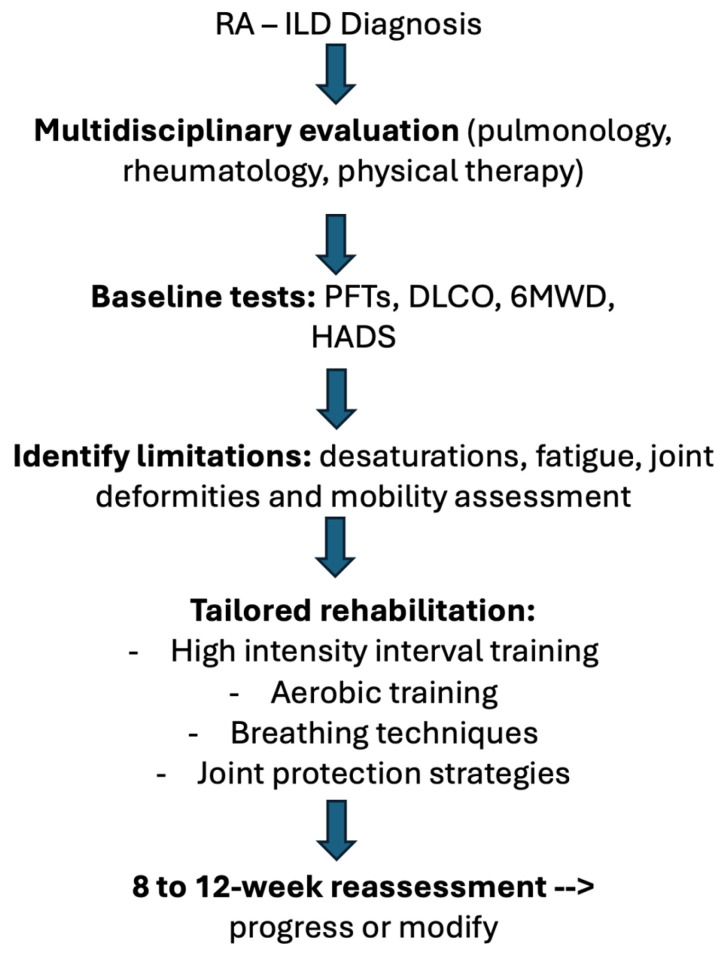
Proposed clinical workflow for implementing individualized rehabilitation in RA-ILD.

Supplemental oxygen is indicated for individuals with exertional desaturation, both to maintain adequate oxygen saturation and to reduce ventilatory drive and dyspnea [22,36,42]. Breathing retraining, pacing, and energy-conservation strategies can further enhance exertional tolerance and symptom control, consistent with PR best practices [16,17]. Figure 2 demonstrates a proposed clinical workflow for implementation.

In individuals with joint pain or structural limitations, cycling, water-based exercise, yoga, and Tai Chi may be advantageous, all of which have demonstrated safety and therapeutic benefit in RA [29,30,31,55].

### 4.5. Safety Considerations and Barriers to Implementation

Safety data from ILD and RA exercise trials strongly support the use of supervised rehabilitation in RA-ILD. PR is consistently reported as safe across ILD populations, including those with advanced fibrosis, with very low rates of adverse events [36,37,38,39,40,45,46,47]. Similarly, RA trials show that aerobic and resistance training do not precipitate joint inflammation, do not increase disease activity, and do not increase flare risk [24,27,52,53,58]. However, it is important to note that most RA exercise trials were conducted in non-hypoxemic patients without clinically significant lung involvement, which limits direct extrapolation of these findings to RA-ILD populations

Nevertheless, RA-ILD patients may require individualized monitoring due to comorbid cardiovascular disease, frailty, osteoporosis, and medication effects such as glucocorticoid-related myopathy [14,15]. Although exercise has not been shown to exacerbate joint disease activity, the risk of acute ILD exacerbation remains a theoretical concern in RA-ILD and warrants close monitoring of oxygen saturation, symptom trajectory, and infection risk during rehabilitation [42].

Barriers to PR participation include limited program availability, transportation challenges, insurance variability, and fear of dyspnea-barriers that mirror those identified in ILD and COPD rehabilitation programs [16,17,48]. In many healthcare systems, including the United States, pulmonary rehabilitation reimbursement is primarily linked to COPD, creating a significant funding barrier for patients with RA-ILD. RA-specific challenges include pain, fatigue, morning stiffness, and variable disease activity. Hybrid and home-based PR models, including tele-rehabilitation, have shown promise for ILD and may improve access for RA-ILD patients [38,48].

### 4.6. Limitations

Several limitations should be considered when interpreting the findings of this review. Most importantly, no randomized controlled trials have specifically evaluated exercise training or pulmonary rehabilitation in rheumatoid arthritis–associated interstitial lung disease (RA-ILD). Consequently, conclusions are derived from indirect extrapolation across three adjacent evidence bases: RA-ILD observational cohorts, pulmonary rehabilitation studies in heterogeneous ILD populations, and exercise interventions in rheumatoid arthritis without significant lung involvement. While this integrative approach is mechanistically plausible, it limits disease-specific inference.

This review was conducted as a structured narrative review rather than a systematic review and therefore does not include a formal risk-of-bias assessment. Although higher-level evidence was prioritized where available, smaller observational and mechanistic studies were included to contextualize findings in this relatively uncommon condition. Additionally, substantial heterogeneity exists across exercise and rehabilitation protocols, including modality, intensity, duration, and outcome measures, limiting standardization and direct comparison across studies.

Most RA exercise trials excluded patients with pulmonary disease or hypoxemia, and pulmonary rehabilitation studies often combined multiple ILD subtypes with limited subgroup reporting for connective tissue disease–associated ILD. As a result, safety and efficacy data may not fully capture the physiologic risks unique to RA-ILD, particularly regarding exertional oxygen desaturation. Furthermore, while exercise has not been shown to exacerbate joint disease or worsen outcomes in broader ILD populations, the risk of acute ILD exacerbation during rehabilitation in RA-ILD remains incompletely characterized, as existing studies were not designed to systematically assess rare pulmonary adverse events.

Finally, publication bias and restriction to English-language studies may have influenced the available evidence. Collectively, these limitations indicate that the conclusions of this review should be viewed as hypothesis-generating rather than confirmatory, underscoring the need for RA-ILD–specific prospective trials.

### 4.7. Future Directions

A major gap in the literature is the absence of randomized controlled trials evaluating PR specifically in RA-ILD. Although CTD-ILD and IPF studies provide substantial insight, RA-ILD has unique immunologic, radiologic, and symptomatic characteristics that warrant targeted investigation [3,13,15,32,33]. Future RCTs should compare PR versus usual care in RA = ILD, stratified by ILD pattern (UIP vs. NSIP) and RA disease activity, with outcomes including exercise capacity, psychological health, oxygen desaturation, acute ILD exacerbations and health-related quality of life.

Mechanistic studies are needed to evaluate skeletal muscle bioenergetics, endothelial function, ventilatory efficiency, and immune modulation in response to PR-domains supported indirectly by RA exercise and ILD rehabilitation literature [5,6,7,27,53,57]. Neural correlates of dyspnea perception, informed by existing neuroscience research [9,10,11], represent another important frontier.

Longitudinal studies should examine whether PR influences hospitalization, exacerbations, health-care utilization, lung function decline, and survival. Implementation science approaches will be crucial in addressing barriers to referral and adherence, evaluating hybrid PR models, and integrating rheumatology-pulmonology rehabilitation pathways into routine care.

## 5. Conclusions

RA-ILD is a progressive and debilitating condition for which non-pharmacologic interventions remain underused despite a strong mechanistic and empirical rationale. Evidence from ILD pulmonary rehabilitation, combined with robust data from RA exercise trials suggests that structured exercise training is likely safe and feasible for patients with RA-ILD, and capable of improving exercise capacity, dyspnea, psychological health, and overall quality of life. Although dedicated RA-ILD rehabilitation trials are lacking, the convergence of mechanistic insights, clinical observations, and cross-disease evidence strongly supports the integration of pulmonary rehabilitation into the routine management of RA-ILD. Future research should focus on RA-ILD-specific clinical trials and explicitly evaluate safety signals, including exertional oxygen desaturation and acute ILD exacerbations, alongside mechanistic investigations to optimize patient outcomes and establish PR as a standard supportive therapy in this high-risk population.

## Figures and Tables

**Table 1:** RA-ILD Observational Cohort Studies.

Study	Sample Size (N)	Study Design	Key Measures	Main Findings
Zamora-Legoff et al., 2017 [32]	N = 167	Retrospective cohort	FVC, DLCO, HRCT pattern	Progressive FVC decline; UIP declines faster; FVC predicts mortality
Hyldgaard et al., 2017 [33]	N = 679	Population-based matched cohort	Comorbidities, mortality	Higher comorbidity burden in RA-ILD patients; ~2× mortality vs. RA alone; UIP has worst survival
Abu Youssef et al., 2015 [34]	N = 45	Cross-sectional observational	UCSD SOBQ, BDI-II, 6MWT, spirometry	Dyspnea strongly associated with depression and reduced functional status
Prioreschi et al., 2014 [35]	N = 38	Prospective observational	Accelerometry, DAS28, inflammatory markers	DMARD initiation ↑ activity and ↓ sedentary time; disease activity improved

**Table 2:** ILD Pulmonary Rehabilitation Trials *.

Study	ILD Type	Sample Size (N)	Study Design	Intervention (+/−Duration)	Outcomes
**Systematic Reviews and Meta-Analyses**					
Dowman et al., 2021 [36]	All ILDs	N = 909 (21 studies)	Systematic review and meta-analysis	PR vs. usual care	PR improved 6MWD, mMRC dyspnea score and HR-QoL In IPF subgroup, outcomes were similar than non-IPF groups
Seleoglu & Demirel, 2024 [37]	CTD-ILD	N = 221 (4 studies)	Systematic review	PR	Moderate evidence for improvement in lung function, DLCO, functional capacity, QoL and dyspnea Limited evidence for respiratory and peripheral muscle strength for CTD-ILD patients
**Randomized and Observational Primary Studies**					
Holland et al., 2008 [38]	Mixed ILD	N = 57 (IPF subgroup = 34)	Prospective multicenter RCT	PR (8 weeks)	Improved 6MWD, dyspnea (Borg and mMRC) and quality of life No significant difference in those with and without IPF.
Holland et al., 2012 [39]	Mixed ILD	N = 44 (IPF subgroup = 25)	Prospective cohort	PR (8 weeks)	Lower baseline function predicted greater PR response Greater improvement in dyspnea and 6MWD with mild IPF, those with other ILDs achieved benefit regardless of severity
Ryerson et al., 2014 [40]	Mixed ILD	N = 54 (IPF subgroup = 22)	Prospective cohort	PR (6–9 weeks)	Improvement in 6MWD, dyspnea and QoL Benefits observed in IPF group and at long-term follow up
Jarosch et al., 2020 [41]	IPF	N = 54	RCT	PR (3 weeks)	Improvement in 6MWD immediately after, HR-QoL and anxiety/depression (HADS)
Vainshelboim et al., 2015 [42]	IPF	N = 34	RCT	Exercise training (12 weeks)	Improved capacity, dyspnea, QoL; fewer hospitalizations
Nolan et al., 2022 [21]	IPF + COPD	N = 326 (IPF subgroup = 163)	Prospective cohort	Structured PR	Improved ISWT distance, dyspnea (MRC score) and quality of life (CRQ) Noncompletion and non-response of PR was associated with higher 1-year all-cause mortality
Wickerson et al., 2018 [43]	Advanced ILD	N = 375 (IPF subgroup = 214)	Retrospective cohort	PR with moderate-intensity aerobic exercise	Patients with advanced ILD required higher levels of supplemental O_2_ to sustain moderate-intensity aerobic exercise Higher O_2_ requirement associated with lower 6MWD and lower intensity training
Gedert et al., 2025 [44]	CTD-ILD	N = 21 (RA-ILD subgroup = 2)	Prospective cohort	PR (8 weeks)	Improved 6MWD, dyspnea and quality of life
Edwards et al., 2023 [45]	IPF	N = 166	Prospective cohort	PR (8 weeks)	Improved mood disorders (depression and anxiety) and functional status
Nishiyama et al., 2008 [46]	IPF	N = 30	RCT	PR (10 weeks)	Improvement in 6MWD and HR-QoL (SGRQ) No statistically significant difference in PFTs and dyspnea
Huppmann et al., 2013 [47]	Mixed ILD	N = 402	Prospective cohort	4-week inpatient PR	Improvement in 6MWD, vital capacity and HR-QoL. No significant difference in dyspnea Patients with pHTN also showed benefit
Güell et al., 2008 [48]	COPD + ILD	N = 51	RCT	Home vs. hospital PR	Improvement in functional exercise capacity and HR-QoL Similar gains across groups
Gazzar et al., 2025 [49]	ILD	N = 80	Prospective interventional cohort	PR (8 weeks)	Improved 6MWD, dyspnea, QoL and some PFTs (FEV1%, FVC and MVV%. No improvement in TLC and DLCO.) Improvement in PaO_2_, SaO_2_%. Decrease in pCO_2_ in PR groups
Wallaert et al., 2018 [50]	Fibrotic IIP	N = 86	Prospective observational cohort	PR	Increased 6MWD and improved anxiety/depression (HADS score)
Brunetti et al., 2021 [51]	Mixed ILD	N = 240 (IPF subgroup = 110)	Prospective cohort	PR	Improved 6MWD, dyspnea, QoL and maximum exercise capacity. No major adverse events

* Several cohorts included patients with CTD-ILD; subgroup-specific outcomes were variably reported and often underpowered.

**Table 3:** RA Exercise Interventions.

Study	Sample Size (N)	Study Design		Intervention	Outcomes
**Systematic Reviews and Meta Analyses**					
**Baillet et al., 2012 [24]**	N = 547 (10 studies)	Systematic review & meta-analysis		Resistance exercise	Improved muscle strength & function; no increase in RA activity; resistance training safe Higher efficacy associated with high-intensity programs Decrease in ESR
**Ye et al., 2022 [52]**	N = 967 (13 studies)	Systematic review & meta-analysis	Aerobic exercise programs	Improved aerobic capacity, fatigue, HAQ; no increase in disease activity or adverse events	
**Stenström & Minor, 2003 [27]**	N/A	Narrative review	Aerobic + strengthening exercise	Exercise improves aerobic capacity & strength; safe without exacerbating RA	
**Randomized and Observational Primary Studies**					
**van den Ende et al., 1996 [53]**	N = 100	RCT		High- vs. low-intensity training (20 weeks)	High-intensity training produced superior strength & functional gains without worsening inflammation
**Lemmey et al., 2009 [54]**	N = 28	RCT		High-intensity resistance training (24 weeks)	Increased lean mass, strength/physical function and IGF-1; no disease flare
**Evans et al., 2011** **[55]**	N = 45	RCT		Iyengar yoga vs. standard care	Improvement in fatigue, mood and chronic pain acceptance Feasible, safe and attractive adjunct treatment
**Wang et al., 2005** **[56]**	N = 20	RCT		Tai Chi, 2×/week for 12 weeks	Improved balance, stiffness, and function; safe with no disease worsening
**Lange et al., 2019 [28]**	N = 74	RCT		Combined aerobic + resistance training, 20 weeks	Improved 6MWD, strength, fatigue, function; well tolerated
**Pukšić et al., 2021 [29]**	N = 60	RCT		Yoga (12 weeks)	Improved pain, fatigue, QoL; no increase in RA activity
**Andersson et al., 2020 [57]**	N = 49	RCT		Moderate-high intensity aerobic + resistance training	Reduced peripheral regulatory T-cell populations; improved fitness; no worsening of disease activity
**Hokkinen et al., 2001 [58]**	N = 70	RCT		Dynamic strength training vs. usual care	Improved muscle strength, function, and bone mineral density; no increase in disease activity

## Data Availability

All data used in this review were derived from previously published studies. No new data were generated.

## Abbreviations

The following abbreviations are used in this manuscript:

AE-ILD	Acute exacerbation of interstitial lung disease
AND	Boolean AND operator
ATS	American Thoracic Society
ATS/ERS	American Thoracic Society/European Respiratory Society
BDI	Beck Depression Inventory
BDI-II	Beck Depression Inventory-II
Borg	Borg Dyspnea Scale
COPD	Chronic obstructive pulmonary disease
CRQ	Chronic Respiratory Questionnaire
CTD-ILD	Connective tissue disease–associated interstitial lung disease
DAS28	Disease Activity Score in 28 joints
DLCO	Diffusing capacity of the lung for carbon monoxide
DMARD	Disease-modifying antirheumatic drug
ERS	European Respiratory Society
ESR	Erythrocyte sedimentation rate
FEV1	Forced expiratory volume in 1 s
FVC	Forced vital capacity
HADS	Hospital Anxiety and Depression Scale
HAQ	Health Assessment Questionnaire
HRCT	High-resolution computed tomography
HR-QoL	Health-related quality of life
IIP	Idiopathic interstitial pneumonia
IGF-1	Insulin-like growth factor 1
ILD	Interstitial lung disease
IPF	Idiopathic pulmonary fibrosis
ISWT	Incremental Shuttle Walk Test
mMRC	Modified Medical Research Council dyspnea scale
MeSH	Medical Subject Headings
MVV	Maximum voluntary ventilation
OR	Boolean OR operator
PaO_2_	Partial pressure of arterial oxygen
pCO_2_	Partial pressure of carbon dioxide
PFT	Pulmonary function test(s)
pHTN	Pulmonary hypertension
PR	Pulmonary rehabilitation
QoL	Quality of life
RA	Rheumatoid Arthritis
RA-ILD	Rheumatoid arthritis arthritis–associated interstitial lung disease
RCT	Randomized controlled trial
SaO_2_	Arterial oxygen saturation
SGRQ	St George’s Respiratory Questionnaire
SOBQ	Shortness of Breath Questionnaire
TLC	Total lung capacity
UCSD SOBQ	University of California, San Diego Shortness of Breath Questionnaire
UIP	Usual interstitial pneumonia
6MWD	Six-minute walk distance
6MWT	Six-minute walk test
